# Plasticity and crosstalk of mesenchymal stem cells and macrophages in immunomodulation in sepsis

**DOI:** 10.3389/fimmu.2024.1338744

**Published:** 2024-01-30

**Authors:** Xingyu Tao, Jialian Wang, Bin Liu, Peifeng Cheng, Dan Mu, Huimin Du, Bailin Niu

**Affiliations:** ^1^ Department of Critical Care Medicine, Chongqing Key Laboratory of Emergency Medicine, School of Medicine, Chongqing University Central Hospital, Chongqing University, Chongqing, China; ^2^ Department of Oncology, The First Affiliated Hospital of Chongqing Medical University, Chongqing, China; ^3^ Department of Surgery, Brigham and Women’s Hospital and Harvard Medical School, Boston, MA, United States

**Keywords:** mesenchymal stem cell, macrophage, immunomodulation, plasticity, crosstalk, sepsis

## Abstract

Sepsis is a multisystem disease characterized by dysregulation of the host immune response to infection. Immune response kinetics play a crucial role in the pathogenesis and progression of sepsis. Macrophages, which are known for their heterogeneity and plasticity, actively participate in the immune response during sepsis. These cells are influenced by the ever-changing immune microenvironment and exhibit two-sided immune regulation. Recently, the immunomodulatory function of mesenchymal stem cells (MSCs) in sepsis has garnered significant attention. The immune microenvironment can profoundly impact MSCs, prompting them to exhibit dual immunomodulatory functions akin to a double-edged sword. This discovery holds great importance for understanding sepsis progression and devising effective treatment strategies. Importantly, there is a close interrelationship between macrophages and MSCs, characterized by the fact that during sepsis, these two cell types interact and cooperate to regulate inflammatory processes. This review summarizes the plasticity of macrophages and MSCs within the immune microenvironment during sepsis, as well as the intricate crosstalk between them. This remains an important concern for the future use of these cells for immunomodulatory treatments in the clinic.

## Introduction

Sepsis, which is characterized by multiple organ dysfunction caused by an imbalanced immune response to infection (1, 2), poses a significant public health challenge due to its high mortality rate and substantial healthcare burden (3). The pathogenesis of sepsis is intricately linked to immune homeostasis, and the early stage is characterized by an excessive systemic inflammatory response, followed by the dynamic interplay between the host immune system and pathogens, which determines the outcome of recovery or prolonged immunosuppression (4, 5). During this process, two types of immune cells play crucial roles.

Macrophages, which are essential components of the innate immune system, are found in the peripheral blood and various organs and contribute to pathogen clearance through phagocytosis. Notably, macrophages exhibit a remarkable degree of plasticity, which is influenced by several factors. Based on their origin, macrophages can be categorized as tissue-resident macrophages (RTMs), which are derived from embryonic tissues, and peripheral blood monocyte-derived macrophages (mo-macs). These subsets undergo distinct differentiation processes, resulting in diverse phenotypes and functions, especially in different immune microenvironments. Park et al. suggest that RTMs mediate persistent inflammatory responses and extensive damage during disease states, while disease-related signals promote the nonsteady-state differentiation of mo-macs, thereby exacerbating disease progression (6). This divergence creates heterogeneity among macrophage subsets. Furthermore, polarization further contributes to macrophage plasticity, and macrophages can be polarized into the M1 and M2 phenotypes by different signalling molecules in the immune microenvironment. These polarized macrophages participate in all stages of sepsis and exhibit nearly opposite immune functions (7). Reports indicate that higher concentrations of circulating cytokines produced by M1-type macrophages are positively correlated with sepsis mortality (8), while M2-type macrophages offer some protection against aggressive inflammatory hyperactivation (9).

Mesenchymal stem cells (MSCs), which are pluripotent stem cells that can differentiate into various cell types, possess the ability to communicate with the inflammatory microenvironment to enhance or suppress the immune system (10). Leveraging the immunomodulatory effects of MSCs by targeting macrophages holds promise for treating sepsis (11, 12). Here, we provide an overview of the immunoregulation mediated by MSCs through different pathways in the context of sepsis. Additionally, we examine the progress made in using MSC-derived extracellular vesicles (MSC EVs) for sepsis treatment, considering their similar biological activities, low immunogenicity, minimal carcinogenic risk, and ability to effectively communicate with other cells by transporting signalling molecules (13, 14).

**Figure 1:**
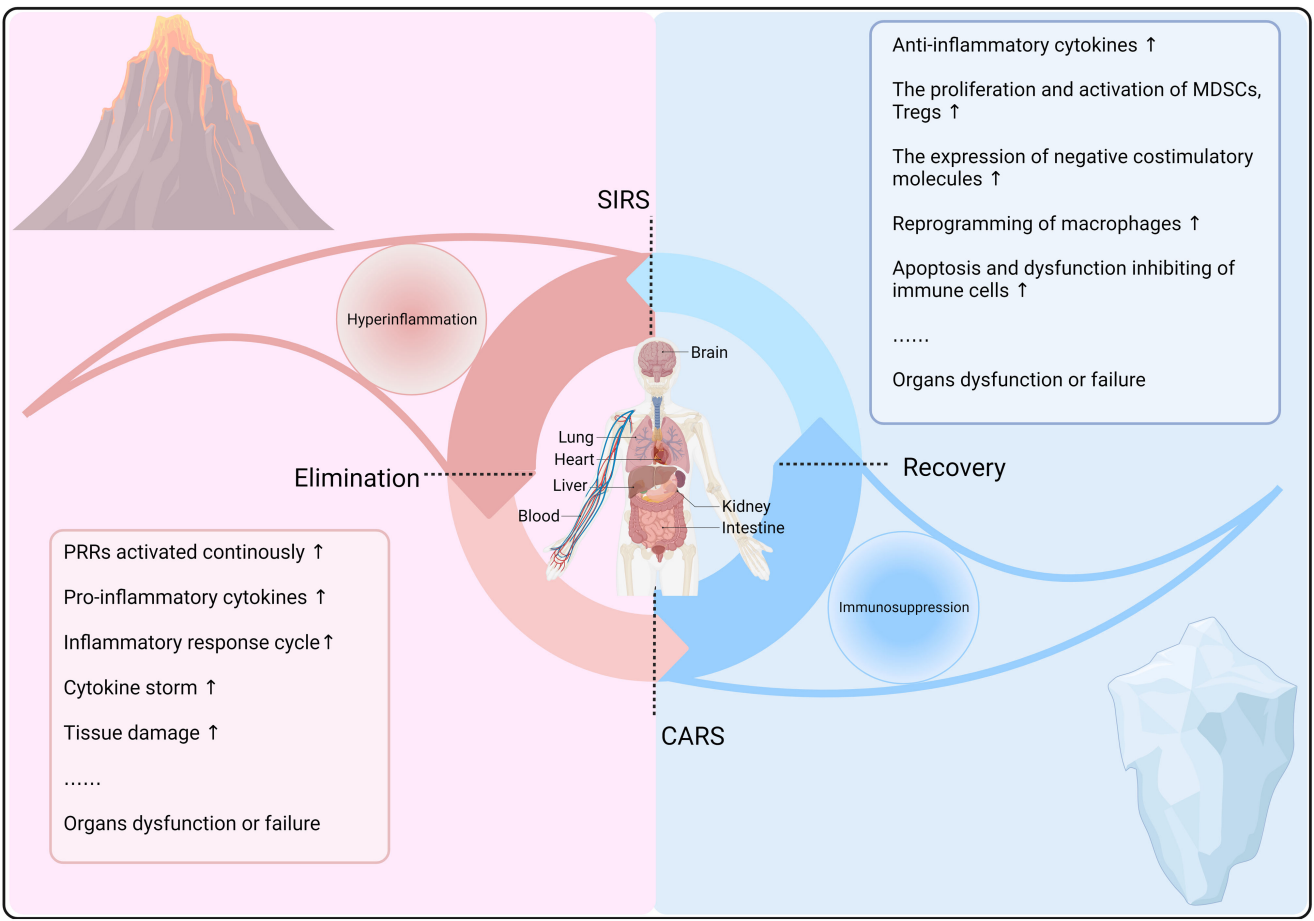
Kinetics of the immune response in sepsis. In sepsis, after the recognition of PRRs by host cells, innate and adaptive immune responses are activated to clear pathogens and may cause a systemic inflammatory response syndrome (SIRS). Concurrently, a compensatory anti-inflammatory response (CARS) prevents excessive inflammation, maintaining immune balance and reducing tissue damage. However, immune dysregulation can lead to a constant state of imbalance, resulting in hyperinflammation or immunosuppression, thereby exacerbating sepsis progression and prognosis.

The development of sepsis is closely connected with the kinetics of the immune microenvironment and immune system regulation (**Figure 1**). Macrophages, with their heterogeneity, plasticity, and functional versatility, play pivotal roles in initiating and regulating the immune response to sepsis. Their ability to clear pathogens, induce tissue damage, promote inflammation, and suppress inflammation can ameliorate or exacerbate sepsis outcomes. Similarly, MSCs have a dynamic association with the inflammatory microenvironment, and alterations in cytokine concentrations within this environment can modulate the immunomodulatory functions of MSCs, subsequently regulating the immune response. In this review, we provide a comprehensive summary of the respective immunomodulatory plasticity of macrophages and MSCs during different immune stages during sepsis, as well as their interactions within the septic environment.

## Basic properties of macrophages

### Sources

Macrophages, which are found in almost all tissues, play a critical role in host defence, wound repair, and immune regulation in mammals. Initially, these cells were classified as part of the monocyte-phagocyte system (MPS) and were believed to be derived solely from monocytes originating from bone marrow precursor cells (15). However, subsequent studies have challenged this view. It has been discovered that the majority of RTMs originate from multipotent erythroid-myeloid progenitor cells (EMPs), which are derived from yolk sac haematopoietic endothelial cells, and haematopoietic stem cells (HSCs) develop from aorta-gonad-mesonephros (AGM) haematopoietic endothelial cells (16, 17). Lineage tracing experiments have further confirmed these findings (18–20). The identification of embryonic-derived macrophages has revolutionized our understanding of the MPS and revealed the existence of diverse macrophage lineages.

### Plasticity of immunomodulatory properties

Recent research has highlighted the importance of considering the differentiation process and dynamics in different environments when studying macrophage ontogeny. It has been proposed that the self-renewal of RTMs *in situ* and monocyte migration and differentiation are the two main sources of the maintenance and expansion of the RTM niche (6). The proportion of RTMs in different organs and tissues tends to change with age (21–26), indicating the necessity to differentiate between subsets and reconsider the functional mechanisms of peripheral blood-derived macrophages. Matthew D. Park and colleagues introduced the concept of nonsteady-state differentiation of monocytes, suggesting that under steady-state conditions, homeostasis-related signals such as M-CSF and GM-CSF contribute to the continuous differentiation of monocytes into RTMs. However, in disease states, disease-associated signals such as interleukin (IL)-33, TSLP, damage-associated molecular patterns (DAMPs), and pathogen-associated molecular patterns (PAMPs) override homeostatic signals, leading to the differentiation of monocytes into disease-associated macrophages (6). Another study demonstrated a similar trend in chronic granulomatous disease (CGD), in which disease progression was promoted by NOX2-producing mo-macs lacking the MHCII^+^CD206^+^CD36^+^ mature phenotype. However, when wild-type (WT) mo-macs and CGD mo-macs were transplanted into CGD mice and WT mice, respectively, their immunophenotype and maturation behaviour were reversed (27). This reversal was attributed to the ability of the inflammatory environment to remodel the phenotype and function of mo-macs.

**Figure 2:**
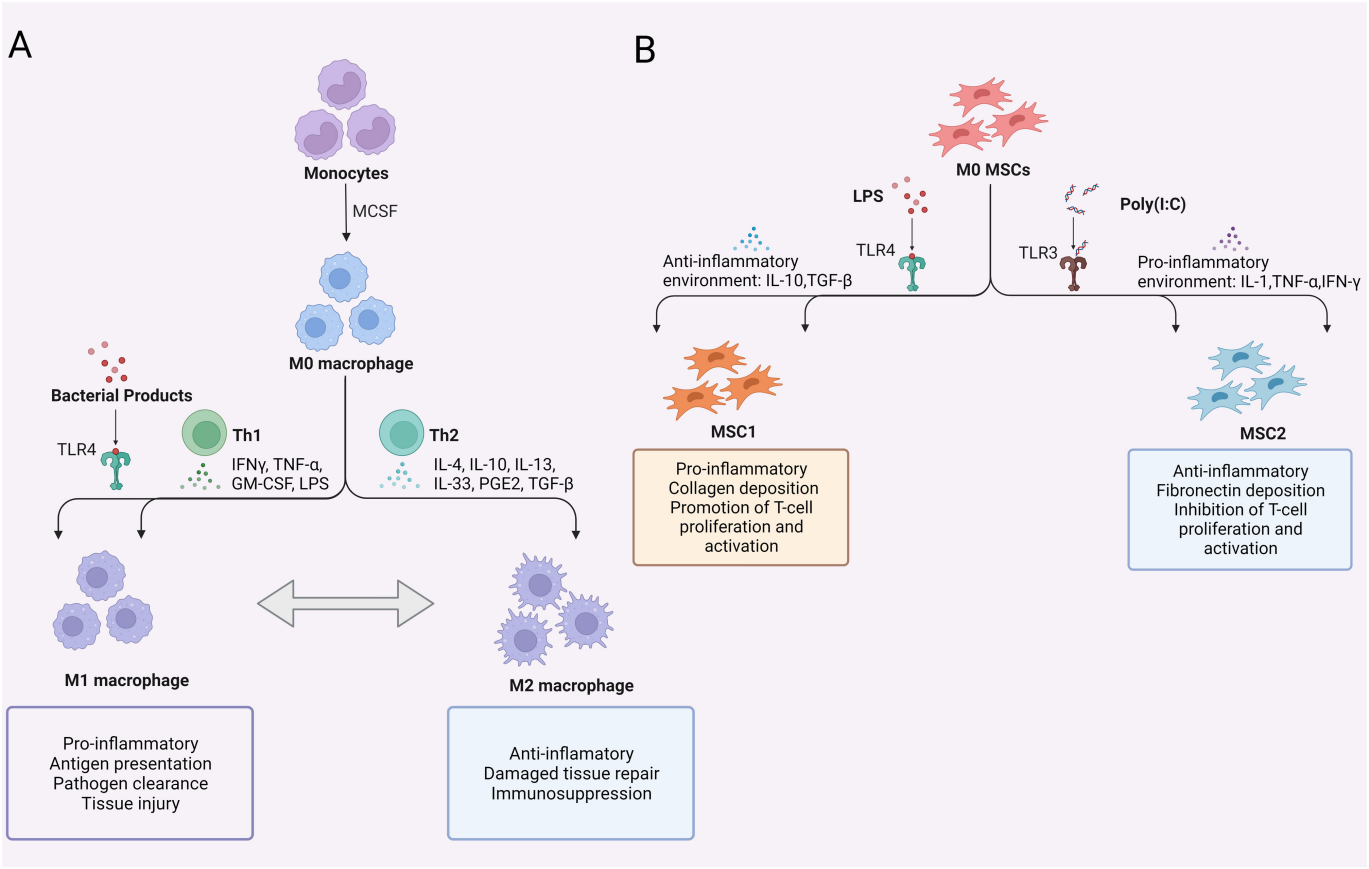
Polarization of macrophages and MSCs. **(A)** Polarization of macrophages; **(B)** Polarization of MSCs. TLR, Toll-like receptor; MCSF, macrophage colony-stimulating factor; GM-CSF, granulocyte-macrophage colony stimulating factor; LPS, lipopolysaccharide; PGE2, prostaglandin-2; TGF-β, transforming growth factor-β; TNF-α, tumor necrosis factor-α; MSCs, mesenchymal stem cells.

Moreover, due to gene expression plasticity, macrophages can be polarized into different phenotypes and perform distinct functions in response to various environmental signals (28). The two main subpopulations are classically activated (M1) macrophages and selectively activated (M2) macrophages (29) (**Figure 2A**). Importantly, macrophage polarization is a dynamic process influenced by multiple environmental factors, and evidence suggests that this process is reversible, suggesting that even polarized macrophages can transition into another phenotype under new immune conditions (30, 31). A growing body of research has shown that macrophage polarization does not accurately and completely capture all phenotypic and functional differences in macrophages, and the heterogeneous functions of macrophages are depending not only on their subgroup but also on the function of the organ or tissue in which the cells are located (32). Therefore, a more detailed understanding of macrophage subtypes with distinct phenotypes and biological functions in different conditions requires further investigation.

## Immunomodulatory plasticity of macrophages during sepsis

### Participation in hyperinflammation during sepsis

During sepsis, macrophages are activated and assume a proinflammatory phenotype in response to the recognition of PAMPs by pattern recognition receptors (PRRs) on the cell surface. These cells are then recruited to the site of inflammation by the chemokines CCL3 and CCL4. Various TLR isoforms can be activated by different pathogenic microbial components. For instance, lipopolysaccharide (LPS) is recognized by the LPS binding protein (LBP), which transfers LPS to CD14 and MD-2 for delivery to macrophages. LPS then binds to TLR4 on the macrophage surface. Subsequently, TLR4 interacts with the MyD88 adaptor protein family, leading to the activation of mitogen-activated protein kinase (MAPK) and the nuclear factor-κB (NF-kB) signalling pathway, thereby inducing a shift in macrophages towards an M1-like phenotype (33, 34).

Activation by pathogens enhances phagocytosis, cytotoxicity, and antigen presentation. Macrophages clear pathogens through oxygen-dependent and oxygen-independent bactericidal systems and initiate innate immune defence responses. Concurrently, these cells recruit other immune cells to jointly increase the production of proinflammatory cytokines such as tumour necrosis factor-alpha (TNF-α), IL-1, IL-6, IL-8, IL-12, and migration inhibitory factor (MIF). Among these, IL-12 activates natural killer (NK) cells and promotes the differentiation of T cells into Th1 effector cells, which produce IFN-γ. IFN-γ enhances the phagocytic and bactericidal functions of macrophages by mediating STAT (35). Studies have indicated that IFN-γ can ameliorate sepsis by increasing the expression of HLA-DR in monocytes and the secretion of TNF-α (36). TNF-α and IL-1, which are released during the early stage of the inflammatory response, are potent proinflammatory mediators. These factors not only reinforce the differentiation of progenitor cells into macrophages and promote macrophage activation but also amplify the inflammatory cascade by activating other immune cells to secrete proinflammatory cytokines. Activated macrophages release MIF and upregulate microbial pattern recognition receptor expression, thereby strengthening the systemic inflammatory response. Previous studies have shown that MIF and CCL2 can cooperatively recruit inflammatory monocytes, leading to high levels of inflammatory factors in septic mice, which can result in lethal shock (37). During the early innate immune defence response triggered by PAMPs, the body promptly responds to the invasion of foreign pathogens. However, sustained release of DAMPs can cause an inflammatory cytokine cascade, triggering a rapid and overwhelming immune response that can lead to systemic response syndrome (SIRS). Notably, the distinction between PAMP and DAMP activation has been highlighted in studies. For instance, HMGB1 can act as a proinflammatory mediator when activated by PAMPs or as a DAMP when passively released from damaged cells during sterile tissue injury. Specific blockade of MD-2 binding to HMGB1 (P5779), which is a tetrameric peptide, without inhibiting the MD-2-LPS-TLR4 signalling pathway has been shown to attenuate DAMP-mediated inflammatory injury (38, 39). Then, activated M1 macrophages upregulate MHC II and the costimulatory molecules CD40, CD86, and CD80 while also secreting CXCL9, CXCL10, and CXCL11 to recruit Th1 cells and initiate the adaptive immune response (40). Moreover, M1 macrophages express high levels of inducible nitric oxide synthase (iNOS) and produce increased levels of NO and reactive oxygen species (ROS), which can damage microorganisms. These macrophages effectively activate and strengthen the immune response to protect the body against exogenous pathogen invasion. Macrophages contribute to the overwhelming immune response during the early stage of sepsis, which inevitably damages tissues and organs. In fact, these cells play dual roles.

### Participation in immunosuppression during sepsis

As sepsis progresses, macrophages can mediate immunosuppression, which is characterized by a decrease in proinflammatory cytokine levels and an increase in anti-inflammatory cytokine production. This transition results in the transformation of M1 macrophages into a distinct immune state known as the M2 phenotype (31, 41). The activation of M2 macrophages is mediated by the Th2 cytokines IL-4 and IL-13, which act through the JAK-STAT6 pathway, as well as IL-10, which uses the JAK-STAT3 pathway. This activation causes Krüppel-like factor (KLF4) and STAT6 to dissociate from the NF-κB coactivator, resulting in the inhibition of NF-κB transcription (7, 42). Furthermore, the binding of IL-13 and IL-33 promotes the accumulation of the hypoxia-inducible transcription factor HIF-2α in M2 macrophages, leading to the upregulation of related genes such as arginase-1 (Arg-1), CCL17, and CCL24 (43). The upregulation of Arg1 and Arg2 expression in M2 macrophages enables the catabolism of arginine into ornithine and polyamines, which supports tissue repair in damaged areas (44, 45). Additionally, M2 macrophages exhibit high expression of the C-type lectin CD206 and the endocytic receptor CD163, enhancing their phagocytic activity and promoting the secretion of CCL17, CCL18, IL-10, transforming growth factor-beta (TGF-β), and IL-1R. This leads to the recruitment of Th2 cells, Treg cells, and other immunosuppressive cells that release significant amounts of anti-inflammatory cytokines. Moreover, M2 macrophages release TGF-β1 and platelet-derived growth factor (PDGF), which promote angiogenesis, tissue injury repair, and the differentiation of fibroblasts into myofibroblasts (46).

Importantly, although these macrophages exhibit marked upregulation of M2 genes, emerging evidence suggests that the reprogramming of these monocytes/macrophages in pathological environments, such as sepsis, differs from classic activation of M2 macrophages (47, 48). For instance, research indicates that P21 plays a crucial regulatory role in programming, and P21 deficiency can lead to an increase in IFN-β. Consequently, the ability of monocytes to be reprogrammed into a hyporesponsive phenotype, which helps avoid inflammatory injury, is compromised (48). Furthermore, several negative regulators of TLR signalling, including MyD88, IRAK-M, and ST2, have been shown to be upregulated in a macrophage tolerance model, resulting in the inhibition of M1 gene expression (49). This phenomenon, which is known as endotoxin tolerance, is characterized by reprogrammed macrophages with a blunted response to endotoxin, unchanged or enhanced release of anti-inflammatory cytokines such as IL-1R and IL-10 and decreased release of proinflammatory cytokines. Davis et al. discovered that impaired wound healing after recovery from sepsis was associated with altered inflammatory phenotypes of monocytes/macrophages. They found that the histone methyltransferase MLL1 could bind to the NF-κB locus, and methylation of the activating histone H3K4me3 altered the monocyte/macrophage phenotype, preventing the initiation of the first stage of wound repair even in the presence of a reduction in inflammatory cytokines. More importantly, studies of bone marrow transplantation have revealed that this epigenetic modification begins in bone marrow progenitor/stem cells and is transmitted to peripheral macrophages, allowing the damage to persist long after sepsis recovery (50). Overall, the reprogramming of macrophage phenotype and function is influenced by immunological homeostasis and the immune microenvironment. There may be a common link between the nonsteady-state differentiation of monocytes mentioned earlier and the endotoxin tolerance observed in reprogrammed macrophages, although further research is needed to establish this connection.

A reduction in the expression of HLA-DR on monocytes/macrophages, which has been associated with impaired monocyte/macrophage function and poor prognosis in sepsis patients, has been observed. In a prospective study, persistent expression of HLA-DR on less than 30% of monocytes was independently associated with mortality in sepsis (51). This downregulation may be linked to the decreased expression of MHC class II molecules and CD86 costimulatory molecules involved in IL-10 and TGF-β signalling, as well as impaired antigen presentation by monocytes/macrophages. Consequently, these cells are unable to bind to T lymphocytes, present pathogen proteins, and activate the adaptive immune response (49). The activation of effector T cells is a critical initial step in the adaptive immune response during sepsis that relies on antigen presentation by macrophages. In addition to their impaired function, macrophages also have increased expression of lymphocyte-negative costimulatory molecules such as PD-L1 and other ligands, thereby negatively regulating the immune response. Increasing evidence suggests a significant increase in T-cell apoptosis during sepsis-induced immunosuppression. Furthermore, the remaining T cells may experience functional exhaustion, which is characterized by impaired production of proinflammatory and anti-inflammatory cytokines, increased expression of negative costimulatory molecules such as PD-1 and TIM-3, and decreased expression of CD127 (IL-7Rα chain) (52, 53). In recent years, progress has been made in targeted therapies for T cells in the context of sepsis (54, 55). *In vitro* experiments using lymphocytes from sepsis patients have demonstrated that treatment with inhibitors of the immune checkpoints PD-1 or PD-L1 can reduce lymphocyte apoptosis (56, 57). Dual blockade of LAG3 and PD-1 has been shown to improve lymphocytic immunosuppression (58, 59).

Therefore, the reprogramming of macrophages from M1-like to M2-like cells, decreasing antigen presentation, and increasing the expression of negative costimulatory molecules are involved in the anti-inflammatory mechanism of sepsis and serve as a compensatory response to the hyperinflammatory state. However, this pathological anti-inflammatory response can lead to immune paralysis, resulting in more severe consequences, such as secondary infection and an increased risk of death (7).

## Basic properties of MSCs

### Sources

MSCs were initially discovered in bone marrow but have since been identified in various tissues, such as bone marrow, fat, lung, heart, and muscle (60, 61). These cells possess the unique ability to self-renew and differentiate into mesoderm-derived cells such as adipocytes, osteoblasts, and chondrocytes, making them a valuable source for tissue regeneration (62). However, the lack of specific markers for MSCs and limited understanding of their *in vivo* biological characteristics pose challenges. Currently, the identification of MSCs relies on nonspecific cell markers (positive for CD29, CD51, CD73, CD90, and CD105; negative for CD31, CD34, and CD45), fibroblast morphology, and tri-lineage differentiation potential (63).

### Multifunctionality

The multipotent differentiation potential of MSCs, combined with their low immunogenicity, safety profile, ease of availability, and ethical acceptance, positions them as promising candidates for stem cell-based regenerative therapies (64–67). The well-established capacity of MSCs to differentiate into various cell types has been extensively reviewed (68–71). Importantly, MSCs obtained from different sources and at different ages exhibit heterogeneous differentiation tendencies and degrees of stemness (72). Additionally, although MSCs lack haematopoietic functions and do not express CD34 (73), they play a unique role in haematopoietic support (74, 75). Perivascular MSCs maintain the self-renewal niche of HSCs by producing stem cell factors (SCFs) and CXCL12 (76). Bone marrow-derived MSCs (BMSCs) promote HSC proliferation and differentiation via the Noch and Wnt signalling pathways (77). A retrospective multicentre study involving 119 patients showed improved survival rates and reduced complications when HSCs and MSCs were cotransplanted (78). Furthermore, MSCs exhibit functional adaptability in tissue injury repair, including promoting angiogenesis, differentiating into myofibroblasts, inhibiting inflammation, and maintaining tissue homeostasis, depending on the type of injured tissue (76).

### Immunoregulatory activity

MSC transplantation has shown potential benefits in treating a wide range of diseases (79–83). However, the large size of MSCs (approximately 15 to 30 μm) makes them prone to entrapment in pulmonary capillaries when administered intravenously, limiting their distribution (84). This suggests the presence of alternative supplementary mechanisms through which MSCs exert therapeutic effects, and the immunomodulatory function of MSCs is believed to play a role in this context (85, 86). MSCs possess chemokine receptors that enable their migration to inflamed tissues, where they regulate the immune response, creating a favourable environment for tissue repair and activating endogenous repair mechanisms (87). It is worth noting that the immunomodulatory ability of MSCs is not constitutive but context-dependent and is influenced by the inflammatory environment. These cells exhibit plastic immunoregulatory functions and exert anti-inflammatory and proinflammatory effects depending on the specific immune microenvironment (88).

## Immunomodulatory plasticity of MSCs during sepsis

### MSC1 and MSC2

Recent studies have shown that MSCs exert rapid immunomodulatory effects that persist even after their depletion, making them advantageous for the treatment of immune-related diseases such as sepsis (83, 89, 90). In the early stage of sepsis, exogenous PAMPs, such as LPS derived from gram-negative bacteria, are recognized by TLR4 on the host cell membrane, and intracellular infection or stress signals activate TLR7 and TLR8 in intracellular host vesicles. Subsequently, the activation of regulatory factors such as MAPK and NF-kB activates immune cells to promote the secretion of inflammatory cytokines, chemokines, and antimicrobial peptides, ultimately initiating the innate immune response to eliminate foreign pathogens. HSP70 has been confirmed to activate the NF-κB signalling pathway through TLR2 on MSCs to induce a proinflammatory phenotype in MSCs (91). TLR4 synergistically recognizes and binds to LPS and has a similar effect (92), while TLR3 activation leads to improved survival in sepsis models (93). MSCs express TLR4 and TLR3, and their activation induces a proinflammatory phenotype (MSC1) or an anti-inflammatory phenotype (MSC2) (94) (**Figure 2B**). The phenotype of MSCs appears to be closely associated with Toll-like receptors, making them potential targets for regulating the immune response in sepsis.

### Immune regulation is affected by inflammatory cytokines

Inflammatory cytokines play a crucial role in stimulating the immunomodulatory activity of MSCs. The upregulation of iNOS or indoleamine 2,3-dioxygenase (IDO) in most mammals serves as the immunosuppressive switch for MSCs during immune regulation in rodents (87). MSCs require activation by IFN-γ and multiple proinflammatory cytokines to upregulate iNOS expression and generate nitric oxide (NO). The secretion of chemokines by MSCs attracts T cells, which are subsequently inhibited by high levels of NO, leading to reduced proliferation and immune activity. In anti-inflammatory conditions, the immune microenvironment is reversed. The expression of iNOS is inhibited, and the secretion of chemokines by MSCs is not reduced, which enhances the immune activity of T cells. Overall, this reversal is influenced by inflammatory cytokines, which can modulate the direction of MSC immune regulation.

### Effects on T lymphocytes

**Figure 3:**
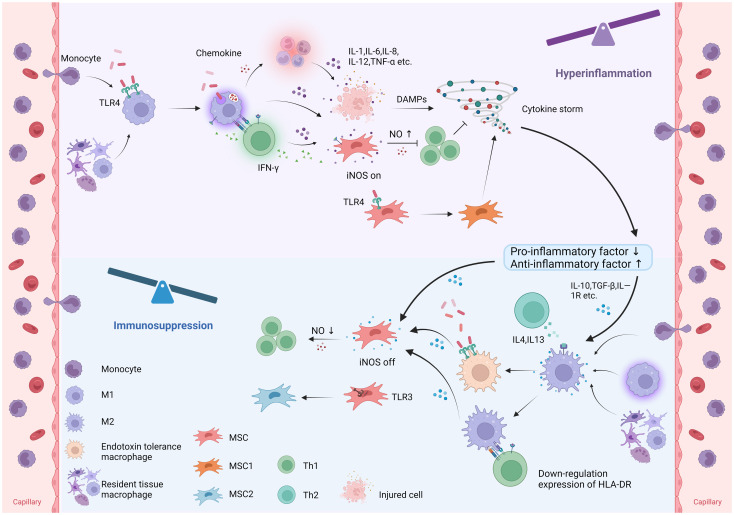
Immunomodulatory plasticity of macrophages and MSCs in sepsis. In sepsis, monocyte-derived macrophages and tissue-resident macrophages polarize to M1 macrophages, while MSCs polarize to MSC1 in response to pathogen recognition. M1 macrophages release proinflammatory cytokines to combat pathogens and induce intense inflammation and tissue injury. MSCs stimulated by proinflammatory cytokines and Th1-derived IFN-γ upregulate iNOS expression to suppress the immune response by inhibiting T-cell function. In addition, MSCs can polarize to MSC1 in response to TLR4 recognition, thereby promoting inflammation. Under anti-inflammatory conditions, macrophages polarize towards the M2 phenotype, while MSCs downregulate iNOS expression, thereby promoting inflammation. Furthermore, MSCs can polarize to MSC2 in response to TLR3 recognition, suppressing inflammation. (TLR, Toll-like receptor; TNF-α, tumor necrosis factor-α; iNOS, inducible nitric oxide synthase; DAMPs, damage associated molecular patterns; HLA-DR, human leukocyte antigen DR; NO, nitric oxide).

T lymphocyte apoptosis is a significant factor in sepsis-induced immunosuppression (95–97). The lymphocyte count serves as an important indicator of early sepsis and is associated with mortality (98, 99), particularly in elderly patients (100, 101). MSCs have emerged as a potential target for regulating the immune response by targeting T cells. Through the secretion of chemokines and adhesion proteins, MSCs recruit immune cells such as T cells (88). In the presence of high levels of inflammatory factors, MSCs directly inhibit T-cell proliferation and differentiation through the production of various immunosuppressive mediators, such as NO, IDO, prostaglandin E2 (PGE2), and TGF-β (102). However, this immunosuppressive effect decreases as the levels of inflammatory cytokines decrease (**Figure 3**). Coculturing MSCs with activated T cells and adding dexamethasone to inhibit T-cell activation has been shown to reduce the production of proinflammatory cytokines and subsequently decrease T-cell immunosuppression (76).

## Crosstalk between MSCs and macrophages during sepsis

### Effect of MSCs on macrophages during sepsis

As previously mentioned, macrophage heterogeneity is determined by their origin, immunophenotype, and tissue specificity. In the context of inflammatory injury, peripheral monocytes migrate to the site of injury and differentiate into various phenotypes based on the homeostatic or nonhomeostatic inflammatory environment. RTMs can also be reversibly polarized into different immunophenotypes and reprogrammed in an inflammatory setting. MSCs can modulate the immune phenotype and function of macrophages through paracrine immunomodulatory mediators, direct cell−cell contact, and the secretion of EVs, thereby restoring immune balance during sepsis.

#### Direct cell-to-cell contact

Based on previous studies, the immunomodulatory effects of MSCs on macrophages are believed to occur through at least three pathways: (1) direct cell-to-cell contact via cell-surface molecules, (2) paracrine signals generated by MSCs such as TGF-β, IDO, PGE2, and TSG-6, and (3) reprogramming in response to phagocytosis by macrophages. Liu et al. established a coculture model of MSCs and CX3CR1-/-Ly6Chi monocytes and found that MSCs upregulated the expression of NR4A1 in proinflammatory Ly6Chi monocytes, transforming them into patrolling Ly6Clo monocytes through direct cell-to-cell contact, thereby improving the prognosis of myocardial infarction (103). In another study on corneal inflammatory lymphangiogenesis, systemic administration of MSCs upregulated the expression of tumor necrosis factor -α-stimulated gene 6 (TSG-6). TSG-6, in turn, interacted with CD44 on the surface of CD11b+Ly6C+ monocytes/macrophages, blocking NF-κB signalling and reducing the recruitment of inflammatory monocytes/macrophages (104). TSG, an ∼35-kDa protein glycoprotein, has subsequently been found to play an important anti-inflammatory role in in a variety of diseases (105). Furthermore, Li et al. demonstrated also that MSCs and proinflammatory macrophages could increase the expression of TSG-6 and CD200 in MSCs through direct contact. This interaction led to the reprogramming of proinflammatory macrophages into anti-inflammatory phenotype, which was facilitated by the binding of CD200 expressed on MSCs and CD200R expressed on proinflammatory macrophages (106). CD200 is another glycoprotein that exhibits anti-inflammatory effects by binding to its receptor, CD200R (107).

#### Paracrine immunomodulatory mediators

MSCs exert paracrine effects through the release of immunomodulatory mediators. One mechanism involves the secretion of chemokines that can recruit nearby T cells by interacting with the T-cell-specific chemokine receptor CXCR3. Additionally, MSCs upregulate the expression of iNOS in an inflammatory environment, leading to the generation of high concentrations of NO. This local increase in NO synergistically inhibits T-cell activity and proliferation (**Figure 3**) (88). Interestingly, a study by Ye et al. demonstrated that overexpression of SIRT1 in MSCs downregulated iNOS expression by reducing the acetylation of NF-κB, while SIRT1-deficient proinflammatory M1 macrophages showed increased iNOS expression in response to LPS activation. The authors suggest that this contradictory pattern may be attributed to the differential effects of iNOS activity on inflammation regulation. The release of NO by MSCs can suppress T-cell proliferation and allogeneic T-cell responses, while proinflammatory macrophages use NO to eliminate invading pathogens (108).

**Figure 4:**
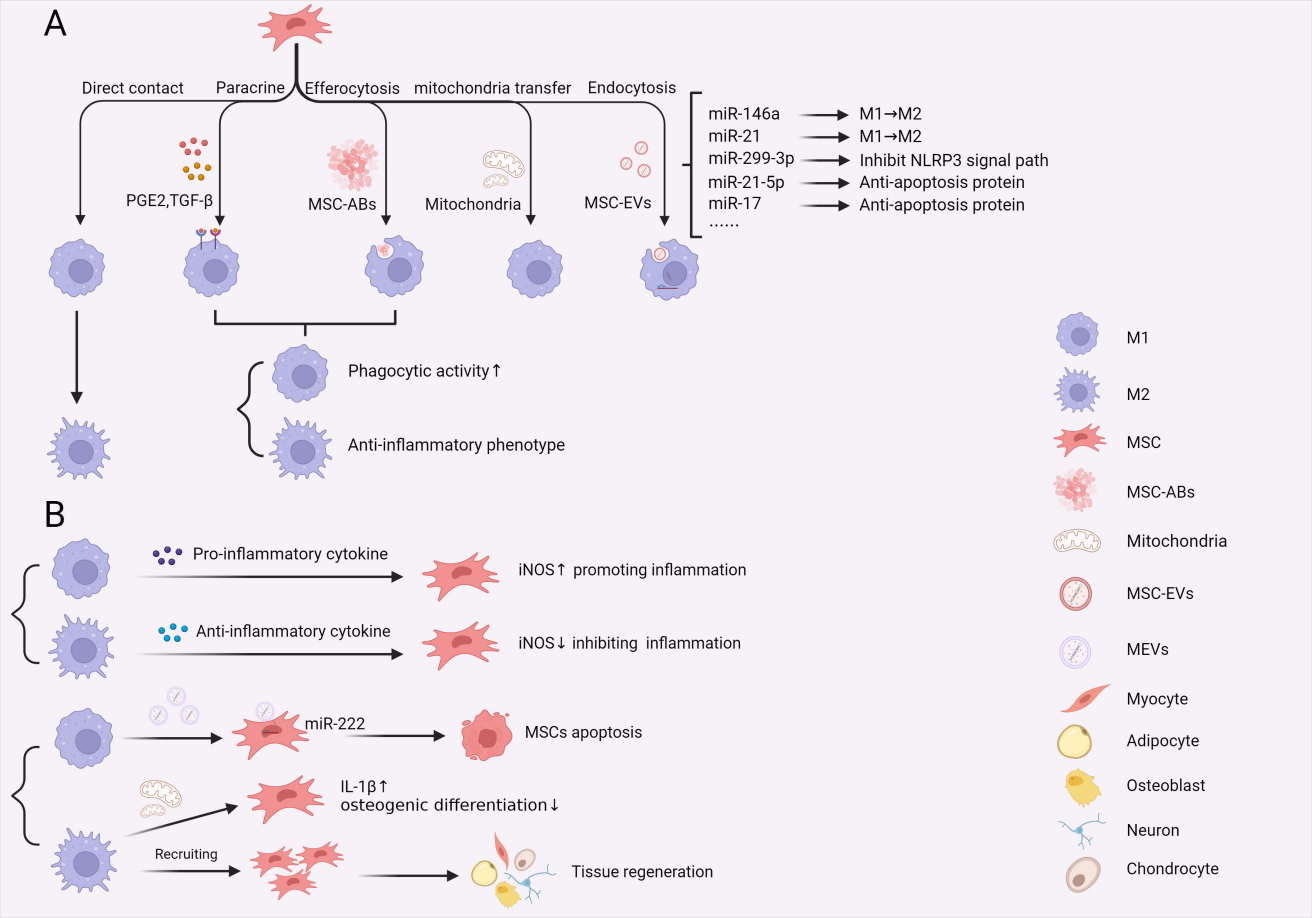
Crosstalk between macrophages and MSCs in sepsis. **(A)** The effects of MSCs on macrophages through direct cell contact, paracrine signalling, efferocytosis, mitochondria transfer and message delivery via MSCs-EVs. These interactions reprogram macrophage phenotype and function during sepsis. **(B)** M1 and M2 macrophages exert different effects on MSCs, including promoting apoptosis, direct recruitment and promoting IL-1β expression, affecting MSC osteogenic differentiation. Additionally, M1 and M2 macrophages modify the immunoregulatory function of MSCs by releasing proinflammatory and anti-inflammatory cytokines, creating distinct immune environments in sepsis. (PGE2, Prostaglandin-2; TGF-β, transforming growth factor-β; MSCs, mesenchymal stem cells; MSC-Abs, mesenchymal stem cell-derived apoptosis bodies; MSC-EVs, mesenchymal stem cell-derived extracellular vesicles; MEVs, macrophage derived extracellular vesicles; iNOS, inducible nitric oxide synthase).

Another important mediator of inflammation that is secreted by MSCs is PGE2 (**Figure 4A**). Following activation by LPS or TNF-α, MSCs upregulate the expression of cyclooxygenase-2 (COX2), which catalyses the release of PGE2. This, in turn, reprograms macrophages to adopt an anti-inflammatory phenotype through the activation of prostaglandin EP2 and EP4 receptors. Consequently, macrophages produce a significant amount of IL-10 and reduced levels of IL-6 and TNF-α (109). Notably, MSCs can induce M1-like and M2-like macrophage phenotypes, and PGE2 release from MSCs can induce an anti-inflammatory phenotype in macrophages while simultaneously enhancing the killing activity of macrophages through increases in NOX2 activity and ROS production (12).

Although numerous studies have confirmed the ability of MSCs to induce an anti-inflammatory M2 phenotype in macrophages for therapeutic purposes (110–112), the traditional “M1/M2 dichotomy” paradigm is being challenged. Increasing evidence suggests that M1 and M2 phenotypes alone are insufficient to characterize the heterogeneity of macrophages, including their regulation by MSCs. One study showed that TGF-β was secreted by MSCs and induced M2 polarization in macrophages while enhancing phagocytosis through the Akt/FoxO1 signalling pathway during the hyperinflammatory phase of sepsis (113). However, an opposing report suggested that the reduction in ROS production in macrophages and suppression of inflammation occurred simultaneously. In a caecal ligation and puncture (CLP) model, BMSCs upregulated the cytosolic E3 ubiquitin ligase (Parkin) expression and increased mitophagy in bone marrow-derived macrophages (BMDMs), thereby reducing mitochondrial ROS production, inhibiting NLRP3 inflammasome activation, and blocking the release of the inflammatory mediators IL-1β and IL-18, thereby inhibiting the inflammatory response (114, 115). Collectively, these findings indicate that MSCs can regulate macrophage phenotypes during different phases of sepsis through distinct mechanisms. MSCs regulate macrophages to adopt an M2-like phenotype to alleviate the inflammatory response during hyperinflammation and an M1-like phenotype to restore immune function during immunosuppression. Furthermore, enhancing the phagocytic and killing activity of macrophages, as well as promoting anti-inflammatory effects and tissue repair, represents a promising strategy for sepsis treatment. Future research should focus on further elucidating the underlying phenomena and mechanisms, identifying specific markers of different macrophage phenotypes, and determining the conditions for their induction. These endeavours will contribute to a comprehensive understanding of the novel mode of MSC-regulated macrophage phenotypes and provide new strategies for the clinical treatment of sepsis using MSCs.

#### Phagocytosis and internalization

Another way in which MSCs exert their effects on macrophages is through internalization and phagocytosis. Macrophage engulfment of MSCs has been investigated in a suppressive model of osteosarcoma, and researchers observed a significant proportion of injected MSCs being engulfed by splenic macrophages before reaching the tumour (116). In an asthma model, MSCs were shown to promote M2 polarization after being engulfed by pulmonary macrophages, thereby improving asthma symptoms (117). Similarly, in a mouse model of sepsis, it was observed that macrophages in the lung phagocytosed MSCs following intravenous injection, leading to increased survival rates in mice (118). However, the question remains regarding how macrophages undergo reprogramming to ameliorate sepsis after phagocytosing MSCs. Min et al. proposed a mechanism whereby macrophages express LRP markers and internalize cytoplasmic components of apoptotic MSCs. Subsequently, these macrophages downregulate genes related to antigen presentation, resulting in the suppression of T-cell activation and reducing inflammation (119). Similarly, Piraghaj et al. demonstrated that macrophages tended to polarize towards the M2 phenotype, leading to decreased production of TNF-α and NO, increased production of IL-10, and enhanced phagocytic activity in response to coincubation with apoptotic MSCs (120). Macrophages possess the ability to perform efferocytosis, which is the engulfment of apoptotic cells, which serves to prevent the release of inflammatory components caused by apoptosis and mitigate inflammatory damage. These findings suggest that the immune activity and function of macrophages change when these cells phagocytose apoptotic MSCs. This may explain the long-term anti-inflammatory effects of apoptotic MSCs after injection and elucidate the interactions between macrophages and MSCs. Additionally, when apoptotic MSCs undergo plasma membrane deformation and form vesicular structures, which are known as MSC apoptosis bodies (ABs), they attract macrophages and undergo phagocytosis due to the exposure of surface signals similar to apoptotic MSCs. Furthermore, MSC ABs can also induce M2 polarization in response to phagocytosis (121) (**Figure 4A**). Therefore, these findings indicate that MSCs not only possess immunomodulatory capacity on their own but that their derivatives can interact with macrophages to regulate the immune response. Further investigations are needed to explore these interactions in more detail.

#### The effects of MSC-EVs on macrophages

Despite the unique advantages of MSCs in the treatment of various diseases, there are still several challenges in their clinical application. First, MSCs tend to be retained in the pulmonary capillaries after injection, which hinders their therapeutic efficacy, and they are rapidly cleared from the body. Second, MSCs can induce immunogenicity during allogeneic transplantation (122). Finally, there is a risk of MSCs migrating to malignant tumours and potentially promoting tumorigenesis (123). In recent years, MSC-derived EVs (MSC-EVs) have emerged as a promising alternative for the treatment of sepsis (124, 125). MSC-EVs exhibit similar biological activities to MSCs and can be categorized into three forms based on their diameter and mode of production: apoptosome ABs (1000-5000 nm), microbubble MVs (100-1000 nm), and exo endosome (40-150 nm). The use of MSC-EVs to treat sepsis has gained increasing attention due to their low immunogenicity and low risk of carcinogenesis, making them safer than cells for transplantation therapy (126). MSC-EVs can be stably present in various body fluids and facilitate intercellular communication by encapsulating and transporting signal molecules such as bioactive molecules, proteins, lipids, and nucleic acids (mRNA, miRNA, and DNA). Numerous *in vivo* and *in vitro* studies have demonstrated that MSC-EVs can deliver these signalling molecules to macrophages through endocytosis or membrane fusion, thereby facilitating immune interactions (127–129). In a pneumonia model, Monsel et al. found that pretreating MSC-EVs with the ILR3 agonist Poly (I:C) enhanced the mRNA expression of COX2, which was then transferred to monocytes. Consequently, the monocytes differentiated into M2 macrophages, leading to reduced production of TNF-α and increased production of IL-10, similar to the effect of MSCs (109, 130). Similarly, in another study, EVs released from adipose-derived MSCs after hypoxic preconditioning were shown to convert macrophages to the M2 phenotype that expressed CD206, CD51, and CD36 *in vitro* (129).

MicroRNAs (miRNAs) have been identified as crucial regulatory substances present in MSC-EVs that exert multiple effects on apoptosis, tumour distribution, and angiogenesis. Over 150 miRNAs have been identified in MSC-EVs, and increasing research has focused on their immunoregulatory effects on macrophages (124) (**Figure 4A**). For example, Li et al. conducted an innovative study demonstrating that MSC-EVs could target and regulate the immunophenotype of macrophages. Through the binding of platelets to monocytes, MSC-EVs can target and bind to peripheral blood monocytes, allowing them to reach the inflammatory microenvironment and reprogram proinflammatory macrophages into anti-inflammatory macrophages, thereby facilitating the repair of heart injury (131). Additionally, several studies have been dedicated to the treatment of sepsis. Song and his team reported that MSCs that were pretreated with IL-1β transferred miR-146a to macrophages through exosomes, resulting in M2 polarization and improved survival rates in septic mice (132). Similarly, Yao et al. discovered that miR-21, which was significantly upregulated in MSC-EVs after pretreatment with IL-1β, could promote M2 polarization *in vitro* and *in vivo* (111). Apart from macrophage polarization, other regulatory mechanisms have been explored. For instance, Zhang et al. demonstrated that MSC-EVs that were pretreated with TNF-α upregulated miR-299-3p and inhibited the NLRP3 pathway in macrophages, thereby reducing inflammatory injury in acute liver failure (133). In another study by Li, it was found that MSC-EVs transferred antiapoptotic miR-21-5p to alleviate lung ischaemia/reperfusion (I/R) injury (134). Additionally, Su et al. discovered that miR-17 in BMSC-derived EVs inhibited LPS-induced macrophage apoptosis by mediating BRD4 expression, potentially providing a novel treatment for immunosuppression in sepsis by relieving apoptosis in immune cells (135). These findings underscore the significant immunotherapeutic potential of MSC-EVs in targeting macrophages for sepsis treatment. However, further studies and clinical translation are needed before MSC-EVs can become a new, safer, and more efficient method for sepsis treatment that targets macrophages.

#### The effects of mitochondrial transfer from MSCs to macrophages

Mitochondrial transfer not only maintains tissue homeostasis and normal development but also facilitates the reversal of damaged tissues and restoration of immune homeostasis (136, 137). Current research has highlighted various ways MSCs transfer mitochondria to macrophages, aiding in immune function and homeostasis restoration (137, 138). For instance, Jackson et al. demonstrated that mitochondrial transfer through tunneling nanotube (TNTs) enhanced macrophage phagocytic ability in an ARDS mouse model (118). Phinney et al. reported that MSC-EVs carrying mitochondria increased ATP levels and oxygen consumption rate upon fusion with macrophages (139). However, the understanding of how mitochondrial transfer specifically mediates macrophage immune function remains very limited. From the perspective of macrophage polarization, M1 macrophages exhibit increased glycolysis, proinflammatory enhancements, mROS accumulation, and mitochondrial dysfunction. In contrast, M2 macrophages display higher oxidative phosphorylation, lower glycolytic flux, and increased mitochondrial activity (140). Morrison et al.’s study on ARDS lung injury showed extracellular vesicle-mediated mitochondrial transfer enhancing macrophage oxidative phosphorylation, promoting M2 marker CD206 expression, and improving phagocytic ability (141). Recently, Hwang et al. investigated the role of mitochondrial transfer in polymicrobial cecal slurry sepsis rat, and found that mitochondrial transfer isolated from L6 muscle cells and umbilical cord mesenchymal stem cells helped improve survival and bacterial clearance (142). Therefore, further understanding of the role of MSCs mitochondrial transfer in sepsis, especially its regulatory role and mechanism on macrophages, is expected to provide a new approach for the immunoregulatory treatment of sepsis.

### Effects of MSCs on different macrophages phenotypes

Actually, the heterogeneity of macrophages poses challenges in studying the immunomodulatory effects and mechanisms of MSCs targeting macrophages in the context of sepsis. The incomplete immune phenotyping and functional definition of macrophages contribute to the difficulty, with oversimplified M1/M2 polarization paradigms prevailing. This oversimplification neglects the true and dynamic immunokinetics of macrophages in the immune microenvironment, hindering a comprehensive exploration of the mechanisms by which MSCs regulate immunity targeting macrophages. Macrophage heterogeneity also arises from their diverse origins within the same tissue. Tissue-resident macrophages maintain immune homeostasis and tissue repair under steady-state conditions, supplemented by peripheral blood monocyte-derived macrophages in varying proportions across organs. Distinguishing between these populations becomes crucial during infections or diseases, where a rapid immune response requires recruitment of macrophages from peripheral monocytes. Current disease models often fail to differentiate between these populations, impacting our understanding of their distinct functions. Recent studies attempted to discern the differences between tissue-resident alveolar macrophages (TRAM) and monocyte-derived macrophages (MoAM) by assessing their impact on lung fibrosis post-injury (143). In a severe pneumonia model, Placental mesenchymal stem cell (PMSCs) treatment downregulated TNF-α expression, preventing the recruitment and M1 polarization of bone marrow-derived macrophages (BMMΦ), while preserving the quantity and function of alveolar macrophages (AMΦs). Further investigation revealed that PMSCs treatment promoted M2 polarization in both BMMΦ and AMΦs. But surprisingly, it enhanced antibacterial functions in AMΦs while inhibiting bacterial phagocytosis and reactive oxygen species (ROS) production in BMMΦ (144). This innovative study underscores the immunoregulatory differences of MSCs on tissue-resident AMΦs and recruited BMMΦ populations. As the concept of non-steady-state differentiation of monocyte-derived macrophages emerges (6), future research should precisely define disease-related mo-macs, explore common pathways across diseases, unravel conservative molecular programs in disease participation, and elucidate specific mechanisms of MSCs targeting mo-macs. Furthermore, clarifying the temporal or spatial relationship between steady-state and non-steady-state differentiation of monocyte-derived macrophages and M1/M2 polarization of macrophages will further enhance our understanding and regulation of MSCs’ immunomodulatory pathways and mechanisms targeting distinct macrophage subpopulations, guiding the direction of MSC therapy for sepsis.

### Effect of macrophages on MSCs during sepsis

The occurrence and progression of sepsis are closely related to immune imbalance, and MSCs possess immunomodulatory abilities. Previous studies have indicated that the ability of MSCs to reduce mortality in mice is dependent on the presence of macrophages, while the depletion of T cells, B cells, or NK cells does not affect the beneficial effects of MSCs (109). Furthermore, the anti-inflammatory effects of MSCs are hindered when macrophages are depleted, as demonstrated by Cutler et al (145). These findings suggest a significant connection between the immunomodulatory effects of MSCs and host macrophages. Consequently, it is crucial to investigate the immune interactions between MSCs and macrophages during sepsis.

#### Effects of macrophages on MSCs by altering the inflammatory microenvironment

During the early stages of sepsis, macrophages are polarized to the M1 phenotype due to the activation of PAMPs and DAMPs. As a result, they release proinflammatory cytokines such as TNF-α and IL-1β, thereby recruiting other immune cells and establishing a hyperinflammatory environment. In this environment, high levels of proinflammatory factors activate the immunosuppressive switch in MSCs, thereby mitigating excessive inflammatory responses (**Figure 4B**). Several studies also emphasized the crucial role of IFN-γ in inducing the anti-inflammatory effects of MSCs (87, 88, 146). Furthermore, IFN-γ produced by activated T cells can directly upregulate PD-L1 expression on MSCs, leading to the inhibition of T-cell proliferation in coculture systems (147), which suggests that the anti-inflammatory effects of MSCs are mediated not only through paracrine signalling but also through direct cell-to-cell contact.

As sepsis progresses into the immunosuppressive stage, macrophages transition towards the M2 phenotype, and the levels of proinflammatory cytokines decrease, resulting in an inability to sustain the hyperinflammatory environment. Instead, there is an increase in the release of anti-inflammatory cytokines that induce MSCs to adopt a proinflammatory phenotype (**Figure 4B**). For example, the administration of IL-10 to MSCs has been shown to enhance T-cell proliferation (148). Additionally, TGF-β acts as an autocrine factor for MSCs and mediates the downregulation of iNOS/IDO expression through the Smad signalling pathway, which inhibits the anti-inflammatory function of MSCs (149). However, most research on the immunoregulatory function of MSCs has focused on the overactivation stage of inflammation during sepsis. Thus, further exploration is needed to elucidate the proinflammatory mechanisms of MSCs during the immunosuppressive stage.

#### Effects of different macrophage phenotypes on MSCs

Macrophages and MSCs are influenced by their surroundings and play bidirectional immunomodulatory roles. While the immune effects of MSCs on macrophages have been extensively studied, the mechanisms underlying the effects of macrophages on MSCs remain incompletely understood. In the microenvironment of acute myocardial infarction (AMI), hypoxia/serum deprivation (H/SD) induces macrophage polarization towards the M1 phenotype, and M1 macrophages induce apoptosis in BMSCs by secreting exosomes containing miR-222, which targets the antiapoptotic protein BCL-2 (150). However, in a mouse subcutaneous implantation model of electrospun poly(ϵ-caprolactone) fibre (PCL-fibre) films, it was found that PCL fibres could recruit M1-type macrophages and promote the transformation of M1-type macrophages to M2-type macrophages, which further enhanced stromal cell-derived factor-1 (SDF-1) secretion and the recruitment of MSCs to the site of injury and played an effective role in tissue regeneration or integration (151). Currently, there is limited evidence on the role and mechanism by which macrophages affect MSCs during sepsis. However, these studies provide insights suggesting that different macrophage phenotypes may have distinct effects on MSCs in different stages of sepsis. Therefore, further investigations are needed to clarify the immune interactions between macrophages and MSCs in different conditions to guide the treatment of sepsis.

#### The effects of macrophage on MSCs by mitochondria transfer

Mitochondrial transfer between cells is a novel communication mode during target cell stress. In a pivotal study, Spees et al. showed that transferring healthy mitochondria can compensate for damaged tissue dysfunction and restore cellular homeostasis (136). It is essential to note that MSCs serve not only as donor cells for mitochondrial transfer but also as recipients from macrophages. MSCs in proliferative or differentiated states exhibit enhanced oxidative phosphorylation (OXPhos) metabolism, increasing mitochondrial activity. Conversely, MSCs with lower proliferative potential shift toward glycolysis, resulting in mitochondrial dysfunction and intracellular Reactive Oxygen Species (ROS) accumulation, diminishing the immunosuppressive function of MSCs (152). Under osteoporotic conditions, Cai et al. found that transferring oxidation-damaged mitochondria from M1-like macrophages to MSCs led to circulating succinate accumulation, increasing ROS levels (153). This activated hypoxia-inducible factor - alpha (HIF-α), promoted IL-1β expression, and affected MSC osteogenic differentiation. In a separate study, transferring mitochondria from one batch of BMSCs to pre-transplantation BMSCs enhanced OXPHOS, ATP production, migration, and osteogenic differentiation in a rat calvaria critical bone defect model. In sepsis, active and damaged mitochondria coexist, impacting MSC immunoregulatory abilities. Macrophages may influence MSC immunophenotypes and functions by transferring mitochondria, though further study is needed to confirm and explore MSCs as mitochondrial transfer recipients in this context.

## Conclusions and perspectives

The pathogenesis of sepsis is intricately linked to the kinetics of the immune response, and proinflammatory and anti-inflammatory responses can coexist. Macrophages, which are crucial innate immune cells, play a significant role in and are influenced by the immune response to sepsis. MSCs possess stem cell characteristics and immunomodulatory abilities, making them important players in sepsis. Additionally, immunomodulation is influenced by environmental factors.

Studies have confirmed the dependency of the immunomodulatory ability of MSCs on the presence of host macrophages. When macrophages are depleted in mice, the anti-inflammatory effect of MSCs are diminished. The activation of inflammatory cytokines is important for upregulating the expression of iNOS in MSCs, thereby triggering the anti-inflammatory switch. Throughout the various stages of sepsis, macrophages polarize into different phenotypes and contribute to changes in the inflammatory environment, indirectly impacting the immunomodulatory effects of MSCs. Limited studies have suggested that different macrophage phenotypes can also directly affect the functional properties of MSCs.

Most studies have primarily focused on the anti-inflammatory effects of macrophages induced by MSCs, and this review outlined the related pathways. First, MSCs reprogram proinflammatory macrophages to an anti-inflammatory state through direct contact, thereby inhibiting NF-kB signalling to reduce the recruitment of inflammatory macrophages. Second, MSCs can influence macrophages via paracrine PGE2 and autocrine TGF-β, directing them towards the M2 phenotype. Notably, MSCs can induce M1-like and M2-like macrophages, resulting in a combination of anti-inflammatory and enhanced phagocytic activity, which may hold potential for sepsis treatment. Consequently, macrophages can be reprogrammed by actively phagocytosing apoptotic MSCs or MSC-derived apoptotic bodies, downregulating antigen presentation, and polarizing towards the M2 phenotype. Finally, MSC-EVs provide a novel cell-free therapeutic approach, as indicated by a recent review that used a meta-analysis to summarize the efficacy of EV treatment of septic animals, which concluded that most MSC-EV treatments reduced mortality in septic mice (125).

In conclusion, macrophages and MSCs exhibit significant plasticity during the progression of sepsis. Their interaction with the immune microenvironment and with each other may hold the key to unravelling the immune imbalance observed in sepsis. Further research is needed to determine the influence of macrophages on MSCs during sepsis and the immune mechanisms of MSCs. The development of precise targeted therapies for patients at different stages of sepsis and the establishment of unified standards are expected to bring positive advancements to sepsis treatment.

## Author contributions

XT: Software, Writing – original draft. JW: Resources, Visualization, Writing – original draft, Software. BL: Methodology, Resources, Writing – original draft. PC: Resources, Software, Writing – original draft. DM: Funding acquisition, Resources, Writing – review & editing. HD: Funding acquisition, Resources, Writing – review & editing. BN: Conceptualization, Funding acquisition, Writing – review & editing.
